# Molecular Mechanisms Underlying Bacterial Uranium Resistance

**DOI:** 10.3389/fmicb.2022.822197

**Published:** 2022-03-10

**Authors:** Tom Rogiers, Rob Van Houdt, Adam Williamson, Natalie Leys, Nico Boon, Kristel Mijnendonckx

**Affiliations:** ^1^Microbiology Unit, Interdisciplinary Biosciences, Belgian Nuclear Research Centre, SCK CEN, Mol, Belgium; ^2^Center for Microbial Ecology and Technology, Ghent University, Ghent, Belgium; ^3^Centre Etudes Nucléaires de Bordeaux Gradignan (CENBG), Bordeaux, France

**Keywords:** reduction, phosphatases, efflux systems, regulation, bioremediation

## Abstract

Environmental uranium pollution due to industries producing naturally occurring radioactive material or nuclear accidents and releases is a global concern. Uranium is hazardous for ecosystems as well as for humans when accumulated through the food chain, through contaminated groundwater and potable water sources, or through inhalation. In particular, uranium pollution pressures microbial communities, which are essential for healthy ecosystems. In turn, microorganisms can influence the mobility and toxicity of uranium through processes like biosorption, bioreduction, biomineralization, and bioaccumulation. These processes were characterized by studying the interaction of different bacteria with uranium. However, most studies unraveling the underlying molecular mechanisms originate from the last decade. Molecular mechanisms help to understand how bacteria interact with radionuclides in the environment. Furthermore, knowledge on these underlying mechanisms could be exploited to improve bioremediation technologies. Here, we review the current knowledge on bacterial uranium resistance and how this could be used for bioremediation applications.

## Introduction

Controlled and accidental releases by nuclear industries, nuclear weapon tests and nuclear accidents have globally spread radionuclides in our environment, including synthetic radionuclides like ^3^H, ^90^Sr, ^131^I, ^137^Cs, and ^241^Am (UNSCEAR, 1988; Van der Stricht and Janssens, 2010; Taira et al., 2013; Prăvălie, 2014; International Atomic Energy Agency [IAEA], 2018). In addition, human activities can lead to an increased exposure to naturally occurring radioactive material (NORM), which primarily include ^238^U, ^232^Th, ^40^K and their decay products (International Atomic Energy Agency [IAEA], 2003). Examples of NORM industries are oil and gas production, coal mining and combustion, metal and uranium mining and processing, geothermal energy production, groundwater treatment, and phosphate mining for fertilizer production (International Atomic Energy Agency [IAEA], 2003; UNSCEAR, 2008). Environmental accumulation of natural and synthetic radionuclides can be hazardous for ecosystems. Transfer of radionuclides to vegetation mainly occurs through water bodies (Salbu et al., 2013), resulting in cytogenetic damage that decreases the reproductive ability (Geras’kin et al., 2013). Furthermore, contamination of water bodies, such as lakes, results in a substantial transfer of radionuclides to fish, herbivores and carnivores (Whicker, 1983; Salbu et al., 2013; Strømman et al., 2013). Humans can be exposed when radionuclides are accumulated through the food chain, through contaminated potable water sources, such as groundwater, or through inhalation (Tompson et al., 2002; Tykva, 2004; Zachara et al., 2013).

Uranium, atomic number 92, is a silvery-white metal belonging to the actinides that is naturally found in minerals such as pitchblende, uraninite, carnotite, autunite, uranophane, davidite and tobernite, but can also occur in phosphate rock, lignite and monazite sands (Lide, 2003). It is one of the principal contaminants of concern in NORM and nuclear industry (NEA/IAEA, 1999; International Atomic Energy Agency [IAEA], 2003). The most common natural isotopes are ^238^U (99.27%), ^235^U (0.72%) and ^234^U (<0.01%). Both ^238^U and ^235^U can be used as nuclear fuel, but ^235^U is more important as it is able to self-sustain a fission chain reaction. Therefore, ^238^U with slightly enriched ^235^U is used for the generation of electricity (Hammond, 2004). ^238^U, ^235^U, and ^234^U decay by emitting an alpha particle and have half-lives of 4.5 × 10^9^ years, 700 × 10^6^ years, and 246 × 10^3^ years, respectively. ^238^U decays 14 times by alpha or beta emission before reaching stable lead-206 (^206^Pb).

**FIGURE 1:**
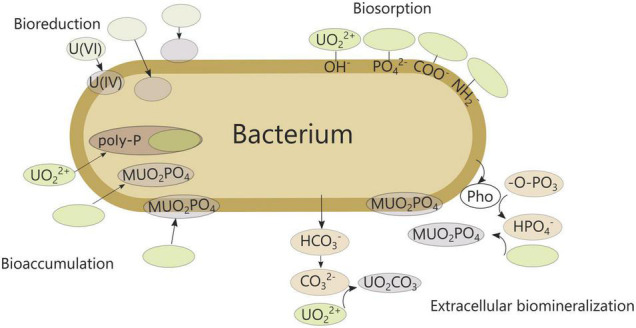
Bacterial interactions with uranium. Uranium minerals are presented as UO_2_CO_3_ or MUO_2_PO_4_ with M indicating a metal ion. Pho, phosphatase; poly-P, polyphosphate granules.

As uranium accumulation could potentially be harmful for humans and ecosystems, strict control and monitoring is essential, and protection and remediation strategies are deployed. Various physical and chemical methods are available, some more advanced than others, but each with its own limitations and drawbacks such as high cost, high complexity and long time span (Godheja et al., 2016). Consequently, there is a need for more simple and ecofriendly alternatives, including biologically based methods. Microorganisms are often found in uranium-contaminated sites and can influence uranium mobility, toxicity and distribution (Choudhary and Sar, 2015). Processes such as biosorption, bioaccumulation, biomineralization and redox transformations are currently well known (Figure 1; Merroun and Selenska-Pobell, 2008). In turn, uranium exerts a permanent pressure on the prevailing microbial population, disrupting microbial communities and processes (Tapia-Rodríguez et al., 2012; Lopez-Fernandez et al., 2017; Sutcliffe et al., 2017). Consequently, fundamental understanding of the interaction between microorganisms and uranium is essential to assess the microbial impact in contaminated environments correctly. Moreover, knowledge on the underlying cellular response can be exploited to improve bioremediation technologies.

The most prevalent oxidation states of uranium in natural environments are U(VI) and U(IV), although it can also exist as U(III) and U(V). Uranium speciation and redox state are important to consider as they influence the mobility of the compound and the toxicity toward biological systems. A large number of factors, such as the aeration state, pH, organic matter, carbonates and phosphates, are able to influence its mobility complicating uranium chemistry (reviewed in Cumberland et al., 2016). In general, the aeration state determines the oxidation state of uranium. Uranyl (UO_2_^2+^) is the main form in oxic systems. Uranyl ions are more mobile and more toxic compared to the reduced uraninite (UO_2_), which can be formed in anaerobic conditions and reoxidized with oxygen (Finch and Murakami, 1999; Markich, 2002; Liu et al., 2017). Also pH determines the solubility of U(VI) and U(IV) complexes. In general, the presence of carbonates has a positive effect on the solubility of U(VI) complexes, especially above pH 5.5. On the other hand, U(IV) is expected to be only slightly soluble in most environmental pH conditions, except at extreme low pH (pH < 3), which can be associated with anthropogenic environments such as acid mine drainage. It is also possible that U(IV) could be mobilized in a colloidal phase. Furthermore, the presence of other ions (e.g., PO_4_^3–^, OH^–^, SO_4_^2–^) and/or organic material can compete for binding to uranyl ions, thereby influencing the mobility of uranium and its sorption to mineral surfaces (Cumberland et al., 2016). Therefore, it is important to consider these factors when investigating microbial interactions with uranium since the toxicity, mobility and interaction strongly depend on the experimental setup.

Although the interaction of microorganisms with uranium is extensively studied, there is far less information about the cellular response of microorganisms to uranium exposure. Data on bacterial uranium resistance mechanisms is rather exploratory. Therefore, instead of discussing the outcome of bacterial interaction with uranium, this review focusses on the different active cellular mechanisms for uranium processing. Microbial reduction of soluble U(VI) to insoluble U(IV) is one of the best-studied mechanisms and many uranium-reducing bacteria have been identified. As such, different reduction mechanisms are discussed in a first section. In a following section, several types of phosphatases are reviewed as metal-phosphate complexation and metal-phosphate biomineralization are common mechanisms for metal detoxification. Afterward, the involvement of membrane proteins, metal efflux and regulatory systems in uranium resistance is reviewed. Finally, the potential application in bioremediation is discussed.

## Uranium Reduction Mechanisms

**FIGURE 2:**
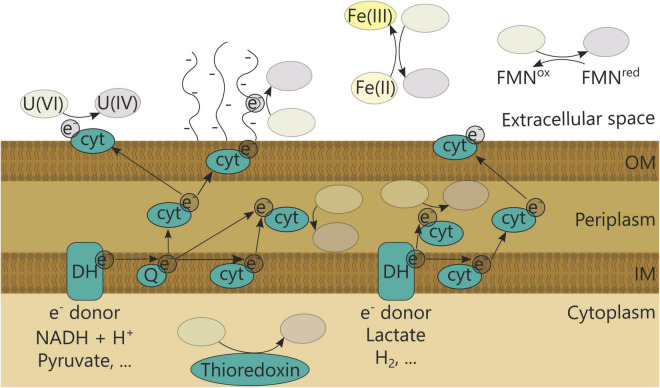
Bacterial uranium reduction mechanisms. DH, Dehydrogenase; Q, quinone; cyt, cytochrome; FMN, flavin mononucleotide.

Enzymatic uranium reduction can occur directly or indirectly in the cytoplasm, periplasm, at the outer membrane or extracellularly (Figure 2 and Table 1) (reviewed in You et al., 2021). It has been investigated particularly in *Geobacter* species, which often dominate in anaerobic uranium-reductive bioremediation setups (Yun-Juan et al., 2005; Shelobolina et al., 2008; Chandler et al., 2010). In general, cytochromes are imperative in the reduction process and were found to be increasingly expressed in *Geobacter uraniireducens* when growing in uranium-contaminated subsurface sediments (Holmes et al., 2009). Moreover, GscA (*Geobacter* subsurface *c*-type cytochrome A) of *Geobacter* sp. M18 was highly abundant during *in situ* uranium bioremediation (Yun et al., 2016). In addition, the diheme c-type cytochrome peroxidase MacA and the outer-surface *c*-type cytochrome OmcZ are essential for uranium reduction, and the periplasmic *c*_7_-type cytochrome PpcA is an important intermediate electron carrier (in the absence of hydrogen) in *Geobacter sulfurreducens* (Lloyd et al., 2003; Shelobolina et al., 2007; Orellana et al., 2013). On the other hand, two outer membrane cytochromes, OmcB and OmcC, showed no or less contribution to U(VI) reduction in *G. sulfurreducens* (Shelobolina et al., 2007). Functional c-type cytochromes were also shown to be essential for uranium reduction in other bacteria. For instance, a cytochrome c maturation deficient mutant of *Shewanella oneidensis* MR-1 was unable to reduce uranium. However, the precise electron transfer pathways involved in uranium reduction are not yet clear. It has been shown that the outer membrane c-type cytochrome MtrC (also known as OmcB), but not OmcA, can function as a terminal uranium reductase. In addition, deletion of both genes decreased the uranium reduction rate and changed the characteristics of the formed uranium nanoparticles (Marshall et al., 2006). A decreased uranium reduction rate was also observed for deletion mutants of *mtrA* (encoding a periplasmic decaheme cytochrome involved in metal reduction), *mtrB* (encoding an outer membrane protein involved in metal reduction) and *menC* (encoding a precursor of menaquinone), which are involved in the electron transfer. In addition, other Mtr-independent pathways can exist (Bencheikh-Latmani et al., 2005). Periplasmic uranium reduction in *Desulfovibrio* occurs mainly via cytochrome c_3_. Deletion of *cycA*, encoding cytochrome c_3_, in *Desulfovibrio alaskensis* G20 (formerly *Desulfovibrio desulfuricans* G20) reduced the uranium reduction rate in the presence of lactate and pyruvate, and almost completely inhibited it with hydrogen gas as electron donor in sulfate-reducing conditions. This indicates that *cycA* is responsible for uranium reduction with hydrogen gas as electron donor, but can be bypassed in the presence of other electron donors (Payne et al., 2002, 2004).

**TABLE 1:** Overview of uranium interaction mechanisms in bacteria.

BACTERIA	Conditions/medium	[U] (mM)	Speciation	Key genes/proteins	References	Comment
**Uranium reduction**						
*Geobacter*sp. M18	*In situ*uranium-contaminated aquifer at ORFRC	NA	NA	GscA?	Yun et al., 2016	Metaproteomic community analysis, no protein with significant similarity to GscA in the genomes of *G. sulfurreducens*, *G. metallireducens*, *G. uraniireducens* or *G. daltonii*

*G. uraniireducens* strain RF4	Heat-sterilized uranium-contaminated sediments	NA	NA	c-type cytochromes	Holmes et al., 2009	/

*G. sulfurreducens*	Fumarate and acetate amended basal bicarbonate buffered medium	1	A	Diheme c-type cytochrome peroxidase MacA	Shelobolina et al., 2007	Decreased U(VI) reduction rate by 98%

*G. sulfurreducens*	Modified freshwater medium	NA	U(VI)	Outer-surface c-type cytochrome OmcZ	Orellana et al., 2013	Approximately 50% less reduction compared to the wild type

*G. sulfurreducens*	Modified freshwater medium	NA	U(VI)	Periplasmic c_7_-type cytochrome PpcA	Lloyd et al., 2003	Depending on the type of electron donor provided, a decrease in reduction is observed

*G. sulfurreducens*	Fumarate and acetate amended modified freshwater medium	1	A	PilA	Cologgi et al., 2011, 2014	Conductive pili

*S. oneidensis*MR-1	Lactate and bicarbonate buffer	0.25	A	CcmC^–^	Marshall et al., 2006	Mutant lacking the ability to covalently incorporate heme into nascent apocytochromes, no U(VI) reduction

*S. oneidensis*MR-1	Lactate and bicarbonate buffer	0.1, 0.25	A	Outer membrane c-type cytochrome MtrC/OmcB	Bencheikh-Latmani et al., 2005; Marshall et al., 2006	Terminal uranium reductase, reduced U(VI) rate

*S. oneidensis*MR-1	Lactate and bicarbonate buffer	0.1, 0.25	A	Outer membrane c-type cytochrome OmcA	Bencheikh-Latmani et al., 2005; Marshall et al., 2006	Reduced U(VI) reduction rate

*S. oneidensis*MR-1	*Shewanella* medium (SM) with lactate and bicarbonate	0.1	A	Periplasmic decaheme cytochrome mtrA, outer membrane protein mtrB, precursor of menaquinone menC	Bencheikh-Latmani et al., 2005	Reduced U(VI) reduction rate

*D. alaskensis* G20	Lactate-Sulfate medium	1	A	cytochrome c_3_ CycA	Payne et al., 2002, 2004	Depending on the electron donor provided, U(VI) reduction rates are reduced or completely inhibited

*D. alaskensis* G20	Modified Lactate-Sulfate medium	2	A	Thioredoxin (MreD), Thioredoxin reductase (MreE), Oxidoreductase (MreG)	Li and Krumholz, 2009; Li et al., 2014	/

						
Iron-related response						

*S. elongatus* BDU 130911	ASN III marine synthetic medium	1	A	Siderophores	Rashmi et al., 2013	Uranium stress induced siderophore production, uranium siderophore complexation was confirmed

*M. oleivorans* A9	0.1 M NaCl	0.01	N	Siderophore iron uptake system	Gallois et al., 2018	Uranium induces an iron starvation response

*D. reducens* MI-1	Modified widdel low phosphate (WLP) medium	0.1	A	Ferrous iron uptake and transport proteins, and a transcriptional regulator of the Fur family upregulated	Junier et al., 2011	/

**U-phosphate precipitation in acid conditions**						

*Serratia*sp. N14	Metal challenge solution; MOPS buffer (purified phosphatase); citrate buffer with G2P	1; ± 0.0125 – 0.3, 1	N	PhoN	Macaskie et al., 1994; Jeong and Macaskie, 1995; Jeong et al., 1997	/

*E. coli* DH5α expressing PhoN from *S. typhi*	Citrate, MOPS NaOH, G2P test solution	1	N	PhoN	Basnakova et al., 1998	/

*E. coli* DH5α expressing PhoC from *M. morganii*	Citrate, MOPS NaOH, G2P test solution	1	N	PhoC	Basnakova et al., 1998	/

*E. coli* DH5α expressing PhoN from a *S.* Typhi isolate	Acetate buffer with G2P	0.8	N	PhoN	Appukuttan et al., 2006	/

*D. radiodurans*R1 expressing PhoN from a *S.* Typhi isolate	Acetate buffer with G2P	0.8	N	PhoN	Appukuttan et al., 2006	/

*M. oleivorans*A9	0.1 M NaCl	0.01	N	PhoE	Gallois et al., 2018	Expression coincided with phosphate efflux and showed uranium-phosphate precipitation

*Caulobacter* OR37	M5G minimal medium with B-vitamins and G2P	0.0005	^233^U	/	Morrison et al., 2021	/
						

*Arthrobacter* sp. X34	Simulated groundwater with G3P	0.2	A	/	Beazley et al., 2007; Martinez et al., 2007	/

*Bacillus* sp. Y9-2	Simulated groundwater with G3P	0.2	A	/	Beazley et al., 2007; Martinez et al., 2007	/

*Rahnella* sp. Y9602	Simulated groundwater with G3P	0.2	A	/	Beazley et al., 2007; Martinez et al., 2007	/

*Rahnella* sp. Y9602	Simulated groundwater with G3P with NO_3_^–^	0.2	A	/	Beazley et al., 2009	/

**U-phosphate precipitation in acid and alkaline conditions**						

*Serratia*sp. strain OT II 7	Acetate (pH 5) or MOPS buffer (pH 7 and 9)	1	N, C	/	Chandwadkar et al., 2018	/

*Chryseobacterium* sp. strain PMSZPI	Acetate (pH 5) or MOPS buffer (pH 7 and 9)	1	N, C	/	Khare et al., 2020	/

**U-phosphate precipitation in alkaline conditions**						

*Sphingomonas*sp. BSAR-1	Carbonate-bicarbonate buffer with G2P	0.5 – 5	C	PhoK	Nilgiriwala et al., 2008	/

*E. coli*BL21 expressing PhoK from *Sphingomonas*sp. BSAR-1	Carbonate-bicarbonate buffer with G2P	0.5 - 5	C	PhoK	Nilgiriwala et al., 2008	/

*E. coli* DH5α expressing PhoK from *Sphingomonas*sp. BSAR-1	MOPS buffer with/without carbonate (pH 9 and 6.8, respectively) with G2P	1	N, C	PhoK	Kulkarni et al., 2016	/

*E. coli* DH5α expressing PhoN from *S.*Typhi	MOPS buffer with/without carbonate (pH 9 and 6.8, respectively) with G2P	1	N, C	PhoN	Kulkarni et al., 2016	/

*D. radiodurans* R1 expressing PhoK from *Sphingomonas*sp. BSAR-1	MOPS buffer with G2P	1 - 10	C	PhoK	Kulkarni et al., 2013	/

*D. radiodurans* R1 expressing PhoK from *Sphingomonas* sp. BSAR-1	MOPS buffer with/without carbonate (pH 9 and 6.8, respectively) with G2P	1	N, C	PhoK	Kulkarni et al., 2016	/

*D. radiodurans* R1 expressing PhoN from *S.* Typhi	MOPS buffer with/without carbonate (pH 9 and 6.8, respectively) with G2P	1	N, C	PhoN	Kulkarni et al., 2016	/

*C. crescentus*NA1000	PIPES buffer (pH 7) with G2P	0.5	N	PhoY	Yung and Jiao, 2014	/
						

*P. rhodesiae R1.2 + P. veronii V1.2*expressing PhoA from *E. coli*	Sterilized soil slurries amended with glycerol 3-phosphate	0.02	A	PhoA	Powers et al., 2002	/

*S. bentonitica*BII-R7	Tris minimal medium amended with G2P	0.1; 0.25	N	Phosphatases?	Pinel-Cabello et al., 2021	Increased induction of four phosphatases

Phytases						

Microbial communities from ORFRC	Sediment slurries	NA	NA	Acid phytase activity	Salome et al., 2017	/

*C. crescentus*CB15N	M2G/M5G minimal medium	0.2 - 1	N	Phytase?	Hu et al., 2005; Yung et al., 2014	Putative phytase was the most highly upregulated protein, involved in uranium resistance when phytate is the only phosphate source

Phosphate release from cellular phosphate sources						

*P. aeruginosa*	MOPS minimal medium	1	N	Polyphosphate kinase	Renninger et al., 2004	Phosphate release from polyphosphate

*D. radiodurans*	0.1 M NaCl	± 0.336	N	/	Suzuki and Banfield, 2004	Phosphate release during cell lysis

*A. torulosa*	Nitrogen-supplemented BG-11 medium lacking phosphate	0.1	C	/	Acharya et al., 2017	Alkaline phosphatases liberate Pi from organophosphate substrates

*Paenibacillus*sp. JG-TB8	0.1 M NaClO_4_ pH 2 – 6, oxic + anoxic	0.5 (pH 2, 3, 4.5), 0.05 (pH 6)	N	/	Reitz et al., 2014	Organic bound uranium at pH 2 and 3; uranium-phosphate at higher pH and oxic conditions

*C. metallidurans*NA4	RM medium	0.1	N	/	Rogiers et al., 2021b	PHB-associated uranium-phosphate

Membrane proteins						

*C. crescentus*NA1000	PYE medium with MES buffer	0.25 – 0.275	N	*rsaF_*a*_* and *rsaF*_*b*_	Yung et al., 2015	/

*B. sphaericus JG-A12*	Nutrient broth (8 g/L)	0.9 (pH 4.5)	N	SlfA	Merroun et al., 2005	Cells, native and recrystallized S-layers

*B. sphaericus NCTC9602*	NA	0.9 (pH 4.5)	NA	SlfB	Raff et al., 2002	native and recrystallized S-layers
						

*D. radiodurans*	20 mM MOPS buffer	1	N	Hpi-PhoN	Misra et al., 2021	Cell-free protein extract

*Microbacterium*	0.1 × TSB medium	0.001	N	UipA	Gallois et al., 2021	

*S. bentonitica*BII-R7	Tris minimal medium amended with glycerol 2-phosphate	0.1, 0.25	N	CreD	Pinel-Cabello et al., 2021	

Metal efflux systems						

*C. metallidurans*NA4	RM medium	0.1	N	*sil, cop* and *czc*genes	Rogiers et al., 2021b	/

*Chryseobacterium*sp. strain PMSZPI	Tris buffered medium	0.5	N	CzcA, czcD, cadA	Nongkhlaw and Joshi, 2019	/

*S. bentonitica*BII-R7	Tris minimal medium amended with glycerol 2-phosphate	0.1, 0.25	N	*czcA/cusA*, *czcD, rcnB, mdtAB*	Pinel-Cabello et al., 2021	/

*G. sulfurreducens*	Fumarate and acetate amended basal bicarbonate buffered medium (anoxic)	0.1	A	Three membrane fusion proteins and two outer membrane factors	Orellana et al., 2014	/

*D. reducens*MI-1	Modified widdel low phosphate (WLP) medium	0.1	A	Cadmium- and copper-translocating P-type ATPAse	Junier et al., 2011	/

*M. oleivorans*A9	0.1 M NaCl	0, 0.01, 0.05	N	/	Theodorakopoulos et al., 2015	Evidence for uranium release

*M. oleivorans*A9	0.1 M NaCl	0.01	N	Upregulation of several cation transporters	Gallois et al., 2018	/

*H. noricense*	3 M NaCl	0.01 -0.12	N	/	Bader et al., 2017	Evidence for uranium release

Regulatory systems						

*C. crescentus*CB15N/NA1000	M2G minimal medium/M5G minimal medium with G2P	0.05 – 1	N	*UrcA*, UzcRS + auxiliary regulators, UrpRS	Hu et al., 2005; Park et al., 2017, 2019; Park and Taffet, 2019	/

*M. oleivorans*A9	0.1 M NaCl	0.01	N	ArsR	Gallois et al., 2018	/

*D. alaskensis* G20	Modified Lactate-Sulfate medium	1 - 2	A	cyclic AMP receptor protein (CRP)	Li and Krumholz, 2009	/

*NA, Not Available; G2P, glycerol-2-phosphate; G3P, glycerol-3-phosphate; N, uranyl nitrate; A, uranyl acetate; C, uranyl carbonate.*

Uranium can also be reduced abiotically by iron (Du et al., 2011). However, since cytochromes are often involved in iron reduction pathways (Weber et al., 2006), there could be a link between uranium reduction and iron metabolism. Indeed, siderophores have been shown to form stable complexes with metals and some radionuclides (Bouby et al., 1998; Rajkumar et al., 2010; Rashmi et al., 2013). Hydroxamate-type siderophores were shown to chelate uranium better when complexed with carbonate (Mo et al., 2016), and desferrioxamine-B increased dissolution of uraninite under reducing conditions and could therefore increase uranium mobility (Frazier et al., 2005). In addition, uranium stress induced siderophore production in the cyanobacterium *Synechococcus elongatus* BDU 130911 (Rashmi et al., 2013) as well as a large number of proteins related to iron metabolism in the Chernobyl isolate *Microbacterium oleivorans* A9. The latter include components of the siderophore iron uptake system such as ABC-transport type subunits and siderophore modification enzymes (Gallois et al., 2018). Moreover, uranium exposure evokes an iron starvation response, thereby enhancing the synthesis of iron uptake systems. This is in line with observations in *Desulfotomaculum reducens* MI-1, where several ferrous iron uptake and transport proteins, and a transcriptional regulator of the Fur family were upregulated in the presence of U(VI) (Junier et al., 2011). Although the actual link between iron metabolism and uranium resistance is currently unknown, these observations indicate that siderophores could protect cells from uranium stress through sequestration.

In *Geobacter* species, evidence has also emerged for U(VI) reduction farther from the cell via extracellular pili. *G. sulfurreducens* expressing pili increased the rate and extent of uranium reduction with carbon ligands outside of the cell, while pili-deficient strains precipitated U(IV) mainly in the periplasm (Cologgi et al., 2011). Furthermore, these conductive pili contributed more to uranium reduction than cytochrome OmcZ (Cologgi et al., 2014). This could indicate that the extracellular pili function as a protective mechanism to avoid excessive precipitation in the periplasm, thereby minimizing cytotoxic effects. Furthermore, the metal-chelating properties of rough lipopolysaccharides could complement the extracellular pili by preventing uranium crossing the outer membrane, thereby creating a barrier to maximize extracellular reduction (Clark et al., 2021). However, detailed knowledge about the mechanism is currently lacking. Another form of extracellular U(VI) reduction is through extracellular electron shuttle compounds. The electron shuttle is able to transfer electrons to an electron acceptor in the extracellular environment, such as U(VI), without the direct interaction of cellular compounds with the electron acceptor. This was shown for *Shewanella* species that secreted a flavin mononucleotide, which is able to mediate and accelerate reduction of U(VI) (Suzuki et al., 2010; Yamasaki et al., 2017).

Most studies demonstrated that reduced U(IV) was predominantly localized in the periplasm and at the outside of the cell. Nevertheless, cytoplasmic U(VI) reduction with thioredoxins as electron donor was also found. Transposon mutagenesis studies in *D. alaskensis* G20 showed that the *mre* operon, coding for a thioredoxin (MreD), thioredoxin reductase (MreE) and an additional oxidoreductase (MreG) was essential for uranium reduction (Li and Krumholz, 2009; Li et al., 2014).

Although much research has been performed to elucidate the precise uranium reduction pathway, there is currently no general model that completely explains the electron transport chain during uranium reduction. Moreover, different mechanisms seem to exist in different strains. More research is necessary to reveal the molecular pathways allowing uranium reduction.

## Phosphatases

Metal-phosphate complexation and biomineralization are common mechanisms for limiting metal bioavailability and toxicity (Gudavalli et al., 2018; Zhang et al., 2021), including uranium (Table 1; Wufuer et al., 2017). Since phosphorus is an essential element (Smil, 2000) and soluble phosphate can be scarce in some environments like soil and water bodies, many bacteria use phosphatases to liberate phosphate ions from mineral or organic phosphorus. In fact, organic forms of phosphorus often constitute 30–50% of the total phosphorus (Ruttenberg, 2014). The liberated phosphate ions are able to interact with uranyl, facilitating complexation and precipitation of uranium. Phosphatases, which are either secreted outside the cell or membrane-bound, are broadly categorized based on the pH required for their optimum activity as acid or alkaline. Different sources of phosphate have been used to study phosphatase-mediated uranium-phosphate biomineralization, such as glycerol-2-phosphate (Appukuttan et al., 2006), glycerol-3-phosphate (Powers et al., 2002), phytate (Li et al., 2019) and polyphosphate (Renninger et al., 2004). An overview of the current knowledge of each of these mechanisms is discussed in the following subsections.

### Acid Phosphatase

Enzymatic uranium phosphate precipitation was first observed in *Serratia* sp. N14 (Macaskie et al., 1992), originally classified as *Citrobacter* sp. N14 (Pattanapipitpaisal et al., 2002). Since then, uranium phosphate biomineralization has been shown in different *Serratia* spp. under diverse conditions, including anaerobic conditions and even in the presence of high doses of gamma irradiation (Newsome et al., 2015; Chandwadkar et al., 2018). The periplasmic acid phosphatase PhoN was responsible for uranium complexation as a phosphatase deficient mutant was unable to remove uranium from the growth medium (Macaskie et al., 1994; Jeong et al., 1997). Fragments of the purified PhoN were homologous to PhoN of *Morganella morganii*, *Providencia stuartii*, and *Salmonella enterica* subsp. *enterica* serovar Typhimurium (Macaskie et al., 1994; Jeong et al., 1997). However, uranium removal in those strains was negligible (Macaskie et al., 1994), indicating that additional strain-specific characteristics were needed, e.g., suitable sites for uranium nucleation (Macaskie et al., 2000). While the inner and outer membrane were initially identified as nucleation sites for biocrystallization, further research also proposed initial exocellular nucleation within the lipopolysaccharides aided by supposedly liposome-entrapped acid phosphatases that released Pi in close juxtaposition (Jeong et al., 1997; Macaskie et al., 2000). Moreover, the presence of phosphate-containing extracellular polymeric material putatively provided a protective function and enabled uranium removal (Jeong et al., 1997; Macaskie et al., 2000). Other studies on PhoN-type acid phosphatases of *Serratia* spp. showed that two isoenzymes exhibited a different pH optimum and glycerol 2-phosphate affinity (Jeong et al., 1998). Both isoenzymes were also sensitive to uranyl causing a reduction in their activity (Jeong and Macaskie, 1995).

To explore the role of phosphatases in uranium biomineralization more in detail, several recombinant strains expressing phosphatases have been studied (Basnakova et al., 1998). Although *E. coli* DH5α expressing either *phoN* from *S.* Typhi or the related *phoC* from *M. morganii* exhibited acid phosphatase activity comparable to *Serratia* sp. N14, different uranium removal capabilities were observed. *E. coli* expressing *phoN* exhibited increased removal compared to *Serratia* sp., and both were superior to *E. coli* expressing *phoC* (Basnakova et al., 1998), suggesting different *in vivo* properties of both phosphatases. Putatively, the tertiary structure of PhoN protects the sensitive sites of the enzyme for uranyl ions to access (Basnakova et al., 1998). In addition, heterologous expression of PhoN from a *S.* Typhi isolate in *Deinococcus radiodurans* R1 and in *E. coli* showed comparable uranium removal although a higher phosphatase activity was observed in *E. coli* (Appukuttan et al., 2006). Also, in *M. oleivorans* A9, the expression of a broad specificity phosphatase PhoE coincided with phosphate efflux and showed uranium-phosphate precipitation in different stadia (Gallois et al., 2018).

Acid phosphatase activity and its involvement in uranium biomineralization was also observed in multiple isolates from uranium-contaminated environments. In Jaduguda (India), nine out of twelve strains isolated from uranium mine wastes possessed phosphatase activity (Choudhary et al., 2012). Furthermore, four out of eight isolates from uranium mill tailings pore waters in the region of Limousin (France) possessed acid phosphatase activity, while only one, *Microbacterium oxydans* Br5, possessed alkaline and acid phosphatase activity (Sanchez-Castro et al., 2017). *Caulobacter* sp. OR37, *Rahnella* sp. strain Y9602 and *Bacillus* sp. strain Y9-2 isolated from the Oak Ridge Field Research Center (ORFRC) were phosphatase-positive and removed uranium from growth medium supplemented with organic phosphate in different pH conditions (Beazley et al., 2007; Martinez et al., 2007; Morrison et al., 2021). Moreover, *Rahnella* sp. Y9602 was able to induce uranium phosphate complexation in nitrate-reducing conditions at pH 5.5, albeit with some differences in initial precipitation rates. The same uranium mineral was formed in both aerobic and anaerobic conditions and this was similar to uranium precipitated with free orthophosphate, suggesting that the precipitation is purely chemical through the liberation of Pi from organophosphate by phosphatases (Beazley et al., 2009). This further supports the hypothesis that cells govern nucleation sites for uranium phosphate complexation. Indeed, Morrison et al. (2021) indicated that abiotic precipitation does not occur at uranium concentrations below 1 μM with 500 μM Pi and below pH 5. However, introducing *Caulobacter* sp. strain OR37 resulted in uranium precipitation and the formation of intracellular polyphosphate granules. Presumably, the cells concentrated uranium at the membrane by sorption, which lowered the activation energy required for nucleation and mineralization that prevented abiotic uranium-phosphate mineralization. While most studies investigate uranium biomineralization at uranium concentrations higher than 20 μM, where uranium-phosphate precipitates will inevitable form if Pi is released, many contaminated sites have lower uranium concentrations (Morrison et al., 2021). Moreover, limitations for uranium in drinking water are often below 1 μM (Nolan and Weber, 2015). Therefore, studies investigating microbial interactions with low uranium concentrations are essential.

Results discussed above are mainly from acidic or near neutral environments. However, different observations could be made in alkaline conditions. *Serratia* sp. strain OT II 7, isolated from the acidic sub-surface soil of a uranium ore deposit, exhibited much higher phosphatase activity at pH 5 compared to pH 7 and 9 in the absence of uranium. However, this strain removed uranium much faster at pH 9 and pH 7, than at pH 5 (Chandwadkar et al., 2018). Moreover, for *Serratia* sp. strain OT II 7 as well as *Chryseobacterium* PMSPZI uranium precipitates were formed at a different cellular location depending on the pH. At pH 9, uranyl precipitated always extracellular. At pH 7, extracellular and cell-bound uranium precipitates were formed. Whereas, at pH 5, uranyl precipitates were mainly cell surface-associated or intracellular, which also decreased phosphatase activity and negatively impacted cell viability (Chandwadkar et al., 2018; Khare et al., 2020). It is clear that acid phosphatases play a prominent role in the precipitation of uranium and enable bacteria to withstand high uranium concentrations. Furthermore, uranium-contaminated environments are often acidic, also evidenced by mainly acidic phosphatase activity of environmental isolates.

### Alkaline Phosphatases

Industrial processes can also lead to alkaline uranium waste (Seidel, 1981), for example by using carbonate-based reagents to recover uranium from historical mine waste (Santos and Ladeira, 2011) via soluble and stable uranium-carbonate complexes (Duff et al., 2004). In those environments, uranium phosphate mineralization is not expected, which indicates the need to study the physicochemical conditions to determine possible uranium-phosphate complexation. Uranium precipitation is possible at pH 9 in the presence of excess carbonate if log(HPO_4_^2–^/HCO_3_^–^) > −3 (Zheng et al., 2006). Indeed, the secreted alkaline phosphatase PhoK from *Sphingomonas* sp. BSAR-1 precipitated the supplemented uranyl-carbonate at pH 9 as uranium-phosphate through glycerol-2-phosphate cleavage. The precipitation was even more rapidly at higher uranium concentrations when PhoK was over-expressed in *Escherichia coli* BL21 (Nilgiriwala et al., 2008). This PhoK has also been used to create the recombinant *Deinococcus radiodurans* Deino-PhoK strain, resulting in efficient extracellular uranium-phosphate precipitation in planktonic and alginate-encapsulated state. Precipitation occurred also during high doses of ionizing radiation and in the presence of cesium and strontium, which are often present in intermediate and low level liquid radioactive waste (Kulkarni et al., 2013). The advantages of using *D. radiodurans* in treating radioactive waste have recently been reviewed in Li et al. (2021). Uranium precipitation with PhoK-expressing recombinant *E. coli* DH5α and *D. radiodurans* was also examined in a carbonate-deficient condition at pH 6.8 (GC 1) versus a carbonate-abundant condition at pH 9 (GC 2). Uranium toxicity was clearly higher in GC 1 coinciding with more uranium adsorption to the biomass. Consequently, uranium precipitation was cell-associated in GC 1 for both *E. coli* and *D. radiodurans*, whereas precipitates were located more distant from cells in GC 2 (Kulkarni et al., 2016). These observations corroborate that uranium speciation and toxicity depend on the environmental conditions.

*C. crescentus* NA1000 forms uranium-phosphate precipitates extracellularly or on the cell surface by releasing Pi in modified M5G medium. The periplasmic alkaline phosphatase PhoY, related to the secreted PhoK from *Sphingomonas* sp. BSAR-1 (39% amino acid identity and 51% similarity), was identified as essential in this process and consequently also for uranium resistance (Yung and Jiao, 2014). Furthermore, heterologous expression of the *E. coli* alkaline periplasmic phosphatase PhoA in three *Pseudomonas* subsurface isolates released sufficient Pi in sterilized soil slurries to remove uranium from the cell-free supernatant (up to 69% of 20 μM uranyl acetate) for *P. rhodesiae* R1.2 and *P. veronii* V1.2, but not *P. fluorescens* F1.2 (Powers et al., 2002). In *Stenotrophomonas bentonitica* BII-R7, isolated from Spanish bentonite clay formations, four phosphatases were upregulated in the presences of uranium and facilitated the formation of extracellular uranium-phosphate precipitates (Pinel-Cabello et al., 2021). Overall, bacterial alkaline phosphatases are present in a wide range of species and could result in the precipitation of uranium. In the studies presented here, organophosphate was often provided as glycerol-2- or glycerol-3-phosphate. However, in natural environments, other forms of organophosphate can be present such as phytate, which requires specialized phosphatases to release inorganic phosphate.

### Phytases

Phytate, or inositol hexaphosphate, is a naturally occurring organic phosphate that can be abundant in soils and is the main form of phosphorous storage in plants (Turner, 2006). Depending on the pH, on the amount of phytate present and on the available carbonates, abiotic uranium precipitation is not expected and thus phosphate needs to be released for uranium immobilization (Langmuir, 1978; Oh et al., 2004; Salome et al., 2017; Li et al., 2019). Phytases are a special class of phosphatases that catalyze the sequential hydrolysis of phytate to less phosphorylated *myo*-inositol derivatives and inorganic phosphate (Wyss et al., 1999). Although distinct phytate types exist, histidine acid phytases are mostly identified in microorganisms. Acidic, but not alkaline phytase activity was shown to be present in microbial communities from the uranium contaminated ORFRC. Experiments with sediment slurries at pH 5.5 indicated that uranium enhanced phytase activity but also resulted in the production of intermediate inositol phosphate species, probably due to the inactivation of other phosphatases, with a decrease in uranium solubility (Salome et al., 2017). *C. crescentus* CB15N is able to form calcium-uranium-phosphate precipitates in oligotrophic medium in the presence of inorganic phosphate. Interestingly, a putative phytase was the most highly upregulated protein in response to uranium in these conditions (Hu et al., 2005). The phytase is not essential for uranium resistance in M2G medium nor in rich PYE medium but does seem to enhance survival in the presence of uranium when phytate is provided as sole phosphate source. Furthermore, since the phytase-deficient mutant already showed reduced growth in the absence of uranium, it seems essential for growth but not necessarily for uranium biomineralization (Yung et al., 2014).

### Phosphate Release From Cellular Phosphate Sources

Whereas most studies investigated bacterial uranium-phosphate precipitation by supplementing organic or inorganic phosphate, the possibility of phosphate release from innate phosphate sources for uranium biomineralization is also being scrutinized. In *P. aeruginosa*, overexpression of a polyphosphate kinase resulted in 100 times more accumulated polyphosphate. In the presence of uranyl nitrate, it was shown that uranyl adsorbed initially to the cells and was consecutively precipitated as uranyl phosphate mediated by the release of phosphate from polyphosphate (Renninger et al., 2004). In addition, *D. radiodurans* was shown to precipitate uranium in non-growth conditions at pH 4 without any supplemented phosphate source. Therefore, phosphate had to come from cellular material, such as polyphosphate, which was released during cell lysis (Suzuki and Banfield, 2004). Acharya et al. (2017) also hypothesized that in the cyanobacterium *Anabaena torulosa* alkaline phosphatases liberate Pi from organophosphate substrates released during the decomposition or degradation of cells by uranium. For *Paenibacillus* sp. JG-TB8, uranium was bound by organic phosphate at pH 2 and pH 3 independent on aeration conditions (Reitz et al., 2014). However, uranium seemed to precipitate more as meta-autunite-like uranyl phosphate at higher pH and in oxic conditions, while under anaerobic conditions no mineralization was observed due to decreased Pi release. Nonetheless, the only phosphate sources during the experiment were organic substrates from damaged cells (Reitz et al., 2014).

## Membrane Proteins

The outer surface of many bacteria and archaea is covered by a proteinaceous surface layer (S-layer) that serves multiple functions, including survival in specific niches. Although its precise role in many organisms has not yet been identified (Fagan and Fairweather, 2014), its involvement in uranium biosorption has been shown in multiple bacteria (Table 1; Pollmann et al., 2006; Yung et al., 2015). This is mostly considered to be a passive process, but evidence emerged that bacteria might modulate their cell envelope to become more resistant. In *C. crescentus* NA1000, transposon mutagenesis revealed that the *rsaF*_*a*_ and *rsaF*_*b*_ genes encoding outer membrane transporters conferred uranium tolerance. While RsaF_*a*_ and RsaF_*b*_ are known for exporting the highly abundant S-layer protein RsaA, a role for the S-layer itself and other S-layer transport systems in uranium resistance was excluded. However, RsaF was found to be homologous to TolC and mutation resulted in a decreased resistance to cadmium and tetracycline, suggesting that resistance could be governed by interacting with other translocases/pumps. Furthermore, contrary to deletion of *rsaF*_*b*_ and *rsaA*, deletion of *rsaF*_*a*_ and *rsaF_*a*_F_*b*_rsaA* increased uranium accumulation. However, a role for RsaF in outer membrane integrity, which could have increased uranium accumulation, could not be excluded. Nevertheless, RsaF plays an important role in uranium resistance either via uranium efflux or via protection of the outer membrane integrity (Yung et al., 2015). The SlfB S-layer protein of *B. sphaericus* JG-A12, isolated from a uranium mining waste pile is much more effective in uranium binding than the SlfA S-layer protein of the reference strain *B. sphaericus* NCTC 9602. The different affinity for uranium could be explained by a distinct C-terminal region of both proteins. The C-terminal region of SlfB harbors significantly more serine and threonine residues, which are potential phosphorylation sites. Notably, analysis of the downstream region of *slfA* and *slfB* and comparison with S-layer proteins from other *B. sphaericus* strains indicated the involvement of horizontal gene transfer and genomic rearrangements (Pollmann et al., 2005). Interestingly, S-layer proteins can also be utilized to increase uranium removal. A protein fusion of the S-layer protein Hpi of *D. radiodurans* with PhoN displayed efficient uranium removal (Misra et al., 2021). Furthermore, a uranyl specific biosensor based on the S-layer proteins of *B. sphaericus* JG-A12 was developed (Conroy et al., 2010).

The involvement of other membrane proteins in uranium binding and resistance has also been shown recently. Comparison of four *Microbacterium* species revealed that protein UipA was only present in uranium-tolerant strains and was the most upregulated protein after uranium induction. Moreover, the C-terminal part of this single-pass transmembrane protein has a high uranyl binding affinity. The crystal structure of UipA displayed a tandem of PepSY domains in a swapped dimer with a negatively charged face, responsible for uranium binding (Gallois et al., 2021). In addition, the production of carboxymethyl cellulose modified iron sulfide complex (CMC-FeS) by sulfate reducing bacteria was shown to have increased U(VI) removal capacity compared to chemically produced CMC-FeS because of the presence of extracellular polymeric substances (EPS) containing tryptophan and tyrosine residues (He et al., 2021). Finally, increased expression of CreD, an inner membrane protein from *S. bentonitica* BII-R7, decreased membrane permeability and prevented uranium from entering the cytoplasm, thereby increasing uranium resistance during the lag phase (Pinel-Cabello et al., 2021).

## Metal Efflux Systems

As evidenced in the previous sections, it is clear that bacteria are able to detoxify uranium via different mechanisms. Nevertheless, in some cases the actual process is not directly deducible. For instance, *Arthrobacter* sp. X34, isolated from the ORFRC, did not exhibit phosphatase activity and did not precipitate any uranium, but was equally and even more resistant to uranium than the uranium-biomineralizing *Bacillus* sp. strain Y9-2 and *Rahnella* sp. strain Y9602, respectively (Beazley et al., 2007; Martinez et al., 2007). Another example is the association of uranium-phosphate minerals with polyhydroxybutyrate in *Cupriavidus metallidurans* NA4 (Rogiers et al., 2021b), which is resistant to uranium independent of the presence of *phaC1*, encoding the poly(3-hydroxyalkanoate) polymerase subunit PhaC (Rogiers, 2022). Therefore, other mechanisms could mediate resistance, including metal efflux systems as uranium toxicity is mainly exerted through its chemical metal-related properties.

**FIGURE 3:**
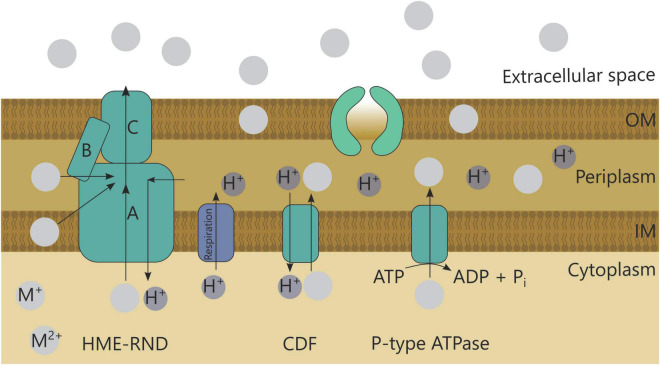
Overview of bacterial efflux systems HME-RND, CDF, and P-type ATPase.

Metal efflux is a common detoxification strategy employed by bacteria. Although multiple systems exist, three systems are most common (Figure 3; Table 1; Nies, 2003). First, the resistance-nodulation-cell division (RND) superfamily includes seven protein families involved in several functions such as transport of hydrophobic compounds and nodulation factors, but also heavy metal efflux (HME-RND). The HME-RND protein (A) is usually combined with a membrane fusion protein (MFP, B) and an outer membrane factor (OMF, C) to form a protein efflux complex that can transport substrates from the cytoplasm, cytoplasmic membrane or periplasm to the outside (Nies, 2003). Typical examples are CzcCBA conferring resistance toward Cd^2+^, Zn^2+^, and Co^2+^ (Mergeay et al., 1985), and CusCBA and SilCBA providing resistance toward Cu^+^, Cu^2+^, and Ag^+^ ions (Munson et al., 2000; Mijnendonckx et al., 2013; Randall et al., 2014). A second export mechanism comprises efflux pumps driven by the proton motive force or potassium gradient known as cation diffusion facilitators (CDF) (Nies, 2003), such as CzcD mediating a small degree of Cd^2+^, Zn^2+^, and Co^2+^ resistance (Nies, 1992; Anton et al., 1999). Lastly, P-type ATPases are able to import and export cations through the hydrolysis of ATP (Nies, 2003). Import is important for essential metals, such as MgtA for Mg^2+^ (Snavely et al., 1989), but ATPases can also detoxify metals through export. Several examples are CadA for Cd^2+^ resistance (Nucifora et al., 1989), ZntA for Zn^2+^ resistance (Beard et al., 1997), and CzcP for Cd^2+^, Zn^2+^, and Co^2+^ resistance (Scherer and Nies, 2009).

Although efflux systems are often upregulated after uranium exposure, a designated efflux system for uranium has not yet been identified. For instance, in *C. metallidurans* NA4 almost all genes (from nine different clusters) involved in the response to and detoxification of silver and copper were upregulated after uranium exposure (Rogiers et al., 2021b). In *Chryseobacterium* sp. strain PMSZPI, isolated from a uranium-enriched environment and in *S. bentonitica* BII-R7 *czcA* and *czcA/cusA* genes were upregulated after uranium exposure, respectively (Nongkhlaw and Joshi, 2019; Pinel-Cabello et al., 2021). The expression of one of the two *czc* gene clusters present in *C. metallidurans* NA4 was also induced after exposure to uranium (Rogiers et al., 2021b). Three membrane fusion proteins and two outer membrane factors assisting HME-RND pumps were also upregulated in *G. sulfurreducens* after uranium induction, amongst which CzcC (Orellana et al., 2014). The expression of another *czc* gene, namely of the CDF encoding *czcD*, was found upregulated in both *Chryseobacterium* sp. strain PMSZPI and *S. bentonitica* BII-R7 (Nongkhlaw and Joshi, 2019; Pinel-Cabello et al., 2021). Furthermore, P-type ATPases are also often found upregulated after uranium exposure, such as *cadA* in *Chryseobacterium* sp. strain PMSZPI (Nongkhlaw and Joshi, 2019) and a cadmium- and copper-translocating P-type ATPAse in *Desulfotomaculum reducens* MI-1 (Junier et al., 2011). Altogether, these results suggest that uranium efflux, if active, occurs through systems conferring resistance to Cd^2+^, Zn^2+^, Co^2+^ or Cu^2+^, and Ag^+^. Other efflux-related genes are sometimes upregulated as well, such as the nickel and cobalt efflux regulator *rcnB* and antibiotic resistance efflux genes (*mdtAB*) in *S. bentonitica* BII-R7 (Pinel-Cabello et al., 2021).

In *M. oleivorans* A9, a fast initial biotic removal of uranium was followed by an active release of U(VI), which was accompanied with phosphate efflux for uranium-phosphate biomineralization. However, this release was only seen at a concentration of 10 μM but not at 50 μM uranyl nitrate. It was therefore hypothesized that higher concentrations of U(VI) could inhibit efflux-mediated resistance or that the influx of uranium into the cell would mask the efflux (Theodorakopoulos et al., 2015). In a follow-up proteomic study, the upregulation of several cation transporters (K^+^, Mn^2+^, Zn^2+^, Mg^2+^, and Co^2+^) and an ABC-type transport system co-occurred with uranium efflux (Gallois et al., 2018). A similar release of uranium after initial biosorption was observed for *Halobacterium noricense* (Bader et al., 2017). The studies on *M. oleivorans* A9 and *H. noricense* could indicate that uranium efflux is possible, but that uranium resistance is a complex process mediated by a combination of different mechanisms. This could explain why, although efflux systems are often upregulated, proof of essential efflux systems for uranium resistance is still lacking. Uranium efflux could contribute to limit uranium entry, but detoxification seems to be mediated by biomineralization, bioreduction or biosorption.

## Regulatory Systems

To sense and respond to environmental changes bacteria deploy different regulatory systems, including two-component systems (TCSs) that are often involved in the response to metals (Chang and Stewart, 1998; Mergeay and Van Houdt, 2015). Typically, the sensor histidine kinase (HK) senses the metal ion, uses ATP to autophosphorylate a conserved histidine residue and transfers the phosphate group to a conserved aspartate residue on the corresponding response regulator (RR) (Chang and Stewart, 1998). On its turn, the phosphorylated RR regulates the expression of metal resistance genes, but often also autoregulates the expression of the TCS. In *C. crescentus* NA1000, two uranium-responsive TCSs have been identified by analyzing transcriptomic and proteomic data after exposure to non-toxic uranium concentrations (Hu et al., 2005; Yung et al., 2014; Park et al., 2017; Park and Taffet, 2019). UrpRS (uranium responsive phytase regulator and sensor, respectively, CCNA_01362 + 01363) was found to regulate a phytase gene (CCNA_01353) that confers uranium resistance when phytate is provided as the sole phosphate source (Yung et al., 2014; Park and Taffet, 2019). UzcRS (CCNA_02842 + 02845), also responsive toward zinc and copper, regulates the expression of *urcA* (uranium response in *caulobacter*, CC3302), the highest uranium-specific induced gene encoding a periplasmic protein with unknown function (Hu et al., 2005; Park et al., 2017). The promoter region of *urcA* contains two m_5 motives specific for uranium induction that are nearly identical to the 18-bp UzcR recognition motif (Hillson et al., 2007). This site contains the partially palindromic half sites 5′-CATTAC-N_6_-TTAA-3′ found in 44 of 57 UzcR binding regions determined by ChIP-seq (Park et al., 2017). Furthermore, deleting *uzcR* or *uzcS* prevented the Zn-, U- and Cu-dependent induction of the promoter region of *urcA* (P*_*urcA*_*). UzcR is thus presumably the regulator of the m_5 motif and could be involved in the direct recruitment of the RNA polymerase holoenzyme since the m_5 motif is located at a common binding site for transcriptional activators, 43 or 53 bp upstream of the transcription start site (Lee et al., 2012). In general, UzcR binds extensively throughout the genome mainly activating genes encoding proteins with a putative signal secretion signal and/or transmembrane domains (52 of 66 genes) such as metallopeptidases, multidrug-resistant efflux (MDR) pumps, TonB-dependent receptors and many proteins of unknown function (Park et al., 2017). The expression of *uzcRS* is modulated by auxiliary regulators and the TCS is part of a complex signaling network (Park et al., 2019). However, deletion mutants of *uzcRS* disputed an essential role in uranium resistance and the manner of uranium sensing is still unclear since UO_2_^2+^, Zn^2+^, and Cu^2+^ displayed different coordination preferences (Haas and Franz, 2009; Park et al., 2017). It is hypothesized that expression of *uzcRS* and *urpRS* is induced indirectly by uranium. Nevertheless, the combination of both TCS systems has been used to develop a whole-cell biosensor for uranium that showed a highly improved selectivity toward uranium compared to the previously designed whole-cell biosensor based on the *urcA* promoter (Hillson et al., 2007; Park et al., 2019; Park and Taffet, 2019).

Upregulation of regulatory systems after uranium induction has also been observed in other bacteria. A negative regulator of stress-induced operons, ArsR (Silver and Phung, 2005), seemed to be less abundant when uranium is internally biomineralized in *M. oleivorans* A9 but more abundant as long as uranium remains extracellularly (Gallois et al., 2018). Interestingly, the TCS UipRS is located upstream the uranium binding protein UipA in multiple *Microbacterium* species, but a role in uranium sensing and resistance has not yet been shown (Gallois et al., 2021). In *D. alaskensis* G20, a cyclic AMP receptor protein (CRP) was found to possibly regulate expression of the *mre* operon for metal reduction, facilitating uranium reduction through thioredoxin (Li and Krumholz, 2009). Finally, ten TCSs are upregulated in *C. metallidurans* after uranium induction of which five are known to be involved in metal resistance (Rogiers et al., 2021b). While extensive work has only been done in *C. crescentus*, there is currently little known on how uranium is sensed and how this results in the transcription of target genes. Further research is therefore necessary, also in other bacteria, to unravel the underlying regulatory mechanisms. An overview of the current knowledge on the different molecular interaction mechanisms is presented in Table 1.

## General Implications for Technological Applications

The toxic characteristics of uranium urged researchers to investigate possible remediation strategies. This led to the discovery that bacteria could be used for uranium bioremediation, which is now one of the most promising bio-based approaches for the remediation of uranium-contaminated sites (Newsome et al., 2014). Uranium reduction is one of the most studied processes. Although the extensive research has already elucidated large parts of the uranium reduction pathway, it is not yet completely clarified. Further research on these pathways could be useful to understand the uranium reduction mechanism completely, but it could also enable full exploitation and modulation of the reduction process for bioremediation applications. However, one of the disadvantages of uranium reduction for bioremediation purposes is that it depends highly on environmental factors as it necessitates reducing conditions and often requires removal of soil compounds (e.g., nitrate) before it can be applied. In addition, one of the remaining problems for *in situ* bioremediation is that reduced uranium is more or less prone to reoxidation depending on the minerals formed. Moreover, the addition of cadmium, a known inhibitor of thioredoxin, showed complete inhibition of uranium reduction in some conditions, which is important to take into account for bioremediation purposes as uranium contaminated soils are very often co-contaminated with metals such as cadmium, zinc and copper (Li and Krumholz, 2009; Bigalke et al., 2017; Lu and Liu, 2018). Furthermore, zinc and copper were also found to completely inhibit uranium reduction when concentrations reached 25 and 15 mg/L, respectively, due to their toxic effects on sulfate-reducing bacteria (Yi et al., 2007). A more extensive screening of different metals can be useful to identify key inhibitors of uranium reduction. Besides metal ions, also uranyl speciation and concentration, pH, temperature, electron donors and acceptors can affect uranium reduction rates (recently reviewed by You et al., 2021). Nevertheless, uranium reduction can still be an asset in several conditions. For example, if *in situ* conditions are anaerobic, providing electron donors could quickly immobilize uranyl by forming uraninite. Also, if the recovery of uranium is necessary during *ex situ* remediation, reduction in column or batch setups could be more favorable, since U(IV) is easily remobilized.

To overcome a number of the limitations for long-term processes, the research focus shifted in recent years to other uranium interaction mechanisms, such as uranium phosphate biomineralization. For instance, if long-term immobilization is preferential or if the removal of uranium without recovery is the main goal, uranium-phosphate precipitation could be the best option. Uranium-phosphate minerals, such as autunite or meta-autunite, have generally low aqueous solubility (Lobeck et al., 2020), are stable over a wide temperature and pH range (Dzik et al., 2017; Wufuer et al., 2017; Gudavalli et al., 2018) and are not prone to remobilization through reoxidation (Williamson et al., 2014; Romanchuk et al., 2020). Abiotic remediation with Pi has been tested, but resulted rapidly in phosphate mineral precipitation not linked with uranium and clogged pore spaces that inhibited further diffusion, which was alleviated by using microbial activity to release Pi continuously from polyphosphates or phytate (Wellman et al., 2006). Phytate has been shown to be more recalcitrant to degradation than other organic phosphates, which may facilitate its migration in contaminated soils and can be advantages for bioremediation purposes (Wellman et al., 2006). However, also uranium phosphate biomineralization still depends on the environmental conditions. Partial protonation of inorganic phosphate starting below pH 4 could hamper abiotic uranyl phosphate mineralization (Hinsinger, 2001). Above circumneutral pH and in the presence of high (bi)carbonate concentrations, highly soluble uranium-carbonate complexes are formed, which can prevent uranyl phosphate precipitation or can solubilize autunite minerals (Pablo et al., 1999; Gudavalli et al., 2018). On the other hand, uranyl-hydroxide formation could allow precipitation (Chandwadkar et al., 2018). Furthermore, strong organic acids, such as oxalate and citrate, interact directly with uranyl and could affect uranium-phosphate biomineralization. However, organic ligands could also promote the conversion of colloidal particles UO_2_(OH)_2_ to free UO_2_^2+^, which could facilitate uranyl phosphate biomineralization. Nonetheless, uranium-phosphate biomineralization was completely inhibited when organic ligands compete with biotic PO_4_^3–^ (Tu et al., 2019). Moreover, in anaerobic nitrate-reducing conditions, the combined toxicity of uranium and produced nitrite after nitrate reduction suppressed growth (Beazley et al., 2009). Even though uranium was still precipitated, presumably due to the early release of Pi before uranium addition or the continued activity of the phosphatases, nitrite accumulation might have implications on the sustainability of the process. Knowledge on compounds preventing uranium-phosphate biomineralization is currently limited to metals. Chromium was able to interfere with uranium biomineralization by PhoN-expressing *D. radiodurans* cells. Furthermore, introducing YieF, which is able to convert Cr(VI) to the less toxic Cr(III), alleviated this problem (Xu et al., 2018). Cd^2+^ and Hg^2+^ are known to affect soil acid phosphatase activity and Hg^2+^, Cu^2+^, and Cd^2+^ are able to inhibit *E. coli* alkaline phosphatase activity (Alnuaimi et al., 2012; Zheng et al., 2019). In general, since phosphatases are known to play a pivotal role, one can hypothesize that inhibition of phosphatase activity also inhibits uranium-phosphate biomineralization.

Overall, it is clear that the physico-chemical environment imposes restrictions on the applied method, especially for *in situ* processes. Since uranium-contaminated sites or often co-contaminated with toxic metals (Sitte et al., 2015; Boteva et al., 2016; Rogiers et al., 2021a), *in situ* bioremediation necessitates the presence of multiple metal resistance mechanisms, which are often present in the indigenous microbial communities thriving in such contaminated sites (Choudhary and Sar, 2015; Agarwal et al., 2020; Rogiers et al., 2021a).

## Conclusion

We provided an overview of the state-of-the-art on active bacterial uranium detoxification mechanisms including uranium reduction, phosphatases, membrane proteins, efflux and regulatory systems. Although extensive work has been done, completely unraveling the molecular mechanistic insights behind uranium resistance and its regulation necessitates further research. Such mechanistic insights can augment bioremediation processes as evidenced throughout this review.

## Author Contributions

TR, KM, and RV contributed to the conceptualization. TR wrote the original draft of the manuscript. KM and RV performed a critical revision of the manuscript. NL, AW, and NB contributed to the manuscript revision, read, and approved the submitted version. All authors contributed to the article and approved the submitted version.

## Conflict of Interest

The authors declare that the research was conducted in the absence of any commercial or financial relationships that could be construed as a potential conflict of interest.

## Publisher’s Note

All claims expressed in this article are solely those of the authors and do not necessarily represent those of their affiliated organizations, or those of the publisher, the editors and the reviewers. Any product that may be evaluated in this article, or claim that may be made by its manufacturer, is not guaranteed or endorsed by the publisher.
